# *In vitro*-*in vivo* Pharmacokinetic correlation model for quality assurance of antiretroviral drugs

**Published:** 2015-09-30

**Authors:** Ricardo Rojas Gómez, Piedad Restrepo Valencia

**Affiliations:** Centro Internacional de Entrenamiento e Investigaciones Médicas (CIDEIM), Cali, Colombia

**Keywords:** antiretrovirals, permeability, dissolution, pharmacokinetics, bioavailability, correlation

## Abstract

**Introduction:**

The *in vitro-in vivo* pharmacokinetic correlation models (IVIVC) are a fundamental part of the drug discovery and development process. The ability to accurately predict the *in vivo *pharmacokinetic profile of a drug based on *in vitro* observations can have several applications during a successful development process.

**Objective:**

To develop a comprehensive model to predict the *in vivo* absorption of antiretroviral drugs based on permeability studies, *in vitro* and *in vivo* solubility and demonstrate its correlation with the pharmacokinetic profile in humans.

**Methods:**

Analytical tools to test the biopharmaceutical properties of stavudine, lamivudine y zidovudine were developed. The kinetics of dissolution, permeability in caco-2 cells and pharmacokinetics of absorption in rabbits and healthy volunteers were evaluated.

**Results:**

The cumulative areas under the curve (AUC) obtained in the permeability study with Caco-2 cells, the dissolution study and the pharmacokinetics in rabbits correlated with the cumulative AUC values in humans. These results demonstrated a direct relation between *in vitro* data and absorption, both in humans and in the *in vivo* model.

**Conclusions:**

The analytical methods and procedures applied to the development of an IVIVC model showed a strong correlation among themselves. These IVIVC models are proposed as alternative and cost/effective methods to evaluate the biopharmaceutical properties that determine the bioavailability of a drug and their application includes the development process, quality assurance, bioequivalence studies and pharmacosurveillance.

## Abstract (es)

**Introducción:**

Los modelos de correlación *In vitro-in vivo* (IVIVC) son parte integral del proceso de investigación y desarrollo de fármacos. La capacidad de predecir con exactitud el perfil *in vivo* a partir de las observaciones *in vitro* tiene diversas aplicaciones durante el desarrollo exitoso de una formulación.

**Objetivo:**

Desarrollar un modelo integral para predecir la absorción *in vivo* de fármacos antirretrovirales con base en estudios de permeabilidad, solubilidad *in vitro *e* in vivo* y demostrar su correlación con la farmacocinética en humanos.

**Métodos:**

Se desarrollaron y validaron las técnicas bioanalíticas para valorar las propiedades biofarmacéuticas de Estavudina, Lamivudina y Zidovudina. Se evaluó las cineticas de disolución, la permeabilidad en monocapas celulares Caco-2 y la farmacocinética de absorción *in vivo* en conejos y voluntarios sanos.

**Resultados:**

Los valores de AUC acumulados en el sistema de células Caco-2, en la disolución y en el modelo animal, fueron correlacionados con los valores de AUC acumulados en el humano. Con lo anterior se demostró una relación directamente proporcional entre los resultados in vitro con respecto a los obtenidos en la fase de absorción tanto en el humano como en el modelo animal.

**Conclusiones:**

Los métodos analíticos y procedimientos aplicados en la IVIVC demostraron las correspondencias directas entre sí, con altos niveles de correlación. Se proponen estos modelos IVIVC como métodos alternativos costo/efectivos para la valoración de las propiedades biofarmacéuticas que determinan la biodisponibilidad, en el desarrollo de productos, en el aseguramiento de la calidad y como pruebas de bioequivalencia en los programas de farmacovigilancia.

## Introduction

The *in vitro-in vivo* pharmacokinetic correlation models (IVIVC) are a fundamental part of the drug discovery and development process. These models can accurately predict the *in vivo* behavior of drugs based on *in vitro* observations, and their applications include: pharmacokinetic properties testing, quality assurance, pharmacosurveillance and quality control during the development and scale up of a formulation. Correlation is defined, according to the United States Food and Drug Administration (FDA), as "a predictive mathematical model describing the relationship between an *in vitro* property of a dosage form and an *in vivo* response". The property of solid dosage forms most commonly applied for *in vitro* development and quality control tests is the rate of dissolution of the drug and it is usually correlated with the concentration or amount of drug absorbed that reach the plasma circulation at certain time 1. Therefore, an IVIVC model based on dissolution tests allows to predict the *in vivo* behavior of some drugs. 

Even though the correlation between *in vitro* dissolution and *in vivo* absorption has been recognized, no many comprehensive models that include complementary properties to improve the predictive capacity have been developed. Comprehensive models are required taking into account the physical, chemical and biological factors involve in the dissolution and absorption of drugs. The physicochemical and biopharmaceutical properties of the drug and the physiology of the routes of absorption have to be taken into consideration. 

The solubility is determined by factors like the percentage of ionized drug, which determines different properties of the compounds, depending on the pH at the site of dissolution. This is very relevant because the human body displays pH gradients, especially in the gastrointestinal tract (GI), this pH gradient leads to differences in the absorption profile depending on the *in vivo* pH 2.

The goal of this study was to develop a comprehensive model to predict the *in vivo* pharmacokinetic behavior of antiretroviral drugs at the absorption phase, based on *in vitro* permeability and solubility measurements. The rationale behind this study is set in the current context where advances in the pharmaceutical sciences, the pharmaceutical industry and the government health agencies have consolidated the quality assessment of pharmaceutical products by *in vivo *bioavailability studies in humans. However, the financial and ethical implications of these studies require the development of alternatives that can substitute the use of humans. These alternatives should consider the physicochemical properties and permeability of the molecules, according to the Biopharmaceutics Classification System (BCS) 3.

## Materials and Methods

### Reagents

The analytical standards and dosage forms of stavudine (d4T), lamivudine (3TC) and zidovudine (AZT) were provided by Humax Pharmaceutical (Medellin, Colombia). Dulbecco's modified Eagle's medium (DMEM) supplemented with L-Glutamine, nonessential amino acids (NEA 100X), Hanks balanced salt solution (HBSS), (N-[2-Hydroxyethyl]piperazine-N'-[2-ethanesulfonic acid]) sodium salt (HEPES), sodium bicarbonate and penicillin/streptomycin 100X were purchase form Sigma-Aldrich. Fetal bovine serum was purchased from Gibco and all the reagents were prepared in accordance with supplier instructions.

###  Dissolution

The kinetics of *in vitro* dissolution were performed following the specifications of the United States Pharmacopeia, using the dissolution apparatus 2 (Pharma Aliance Group, Model TDT- 08L). The rotation was set at 50 rpm, the dissolution media was degassed water at a final volume of 900 mL and a temperature of 37° C ± 0.5. Samples were taken at 10, 20, 30, 45 y 60 min.

###  Caco-2 cells culture

Caco-2 cells (HTB-37) were obtained from the American Type Culture Collection (ATCC). Cells were used between 30 and 50 passes in high glucose (4.5 g/L) DMEM media supplemented with L-Glutamine, 10% fetal bovine serum, 1% nonessential amino acids and 1% penicillin/streptomycin. For the permeability test, 6x10^4^ cells/cm^2 ^were seeded in *transwell *supports with polycarbonate membrane (0.4 µm, 1.12 cm^2^; Corning Costar). The integrity of the monolayers was tested by measuring the Transepithelial Electrical Resistance (TEER) before and after every experiment. Only monolayers with TEER higher than 200 Ω/cm^2 ^were used. 

The apparent permeability coefficient (Papp) was determined from the amount of drug transported per unit of time and was calculated using the following equation:

*Papp*=(∆Q/∆t)/(AxCo)

Where (∆Q/∆t) is the accumulated concentration in the recipient compartment (µmol/sec) versus the change in time, A is the surface area of the support (cm^2)^ and C_0_ is the initial concentration in the donor compartment (µM). The integrity of the system was evaluated using the permeability coefficient (P). The apparent permeability coefficient (Papp) is associated with the permeability coefficient. 

P= (∆Q/∆t) x (Recipient compartment concentration/Transwell support area)

###  Pharmacokinetics in animals

All protocols and procedures were reviewed and approved by CIDEIM Institutional Review Board for research involving animals, in accordance with national and international guidelines for utmost respect and care of animals, recognizing their intrinsic value as living species, as well as deep reflection on the possibilities of replacement, reduction and refinement of techniques and procedures 4-6. In every pharmacokinetic study 10 New Zeeland rabbits were included per group, their weights were between 2.5-3.2 kg. Rabbits were kept at the animal facility under all standard conditions for the handling (20-25° C; 30-70% relative humidity, natural cycle of light/dark), with access to water and food *ad libitum* up to 2 hours before the study and one hour after the administration of the drug as an oral aqueous suspension. Each group received a single dose of stavudine 8.9 mg/kg, lamivudine 20.8 mg/kg or zidovudine 41.7 mg/kg. Two milliliters blood samples were taken from the marginal ear vein, before the administration of the drug and up to 6 hours after administration, at the following time points for lamivudine and zidovudine: 0.0, 0.25, 0.5, 0.75, 1.0, 2.0, 3.0, 4.0 and 6.0 h. For stavudine, samples were collected up to 3 hours after the administration at 0.0, 0.08, 0.17, 0.25, 0.33, 0.5, 0.75, 1.0, 2.0 and 3 h. Samples were centrifuged at 1,500 rpm for 20 min and plasma was analyzed by high performance liquid chromatography (HPLC) for the quantification of each active pharmaceutical ingredient (API). 

###  Pharmacokinetics in humans

The study and the protocols were approved by CIDEIM Institutional Review Board for research, in accordance with the Declaration of Helsinki (October 2008), the International ethical guidelines for research involving human subjects of the Council for International Organizations of Medical Sciences (CIOMS) and the Colombian Ministry of Social Protection Resolution 2378 from 2008. This study was designed as a randomized, open-label, one-way, one sequence and single-dose study. The participants of the study were 18-35 yrs old males, between ±15% ideal weight, healthy according to the physical examination and the laboratory tests (serum biochemistry, hematology, hepatic function, renal function, thyroid function and urinalysis). After explanation of the study risk and procedures, voluntary, informed, signed consent was provided by each participant. Ten volunteers received a single capsule of stavudine 40 mg (Humax Pharmaceutical) and the second group of 9 volunteers received a single combined tablet of lamivudine 150 mg/zidovudine 300 mg (Humax pharmaceutical) with 240 mL of water. Blood samples (8 mL) were collected before and up to 12 h after the administration of the drug, from a catheter maintained with saline solution. The sampling intervals were: 0.0, 0.17, 0.33, 0.50, 0.75, 1.0, 1.5, 2.0, 2.5, 3.0, 4.0, 6.0, 9.0 and 12 h. 

###  Sample analysis

Human and rabbit plasma samples were analyzed using a UPLC apparatus equipped with UV diode array detector (LaChrome Ultra, Hitachi). Samples were injected onto a Chromolith^®^ Performance RP-18 column (100x4.6 mm, Merck), using a mobile phase containing 10 mM ammonium acetate buffer and acetonitrile (96:4, pH 6.0) at 35º C. The flow rate was 1.2 mL/min. Plasma proteins were precipitated with methanol and the procedure was validates according to FDA guidelines 7.

###  Statistical analysis

Pharmacokinetic parameters were calculated using non-compartmental methods, with log transformation of concentration data. The elimination half-life (t1/2) was estimated from the linear regression of the concentration curve versus time. The area under the plasma concentration curve versus time was determined by the trapezoidal method using the PK Solutions Pharmacokinetics Data Non-compartmental Analysis^®^ software (Summit Research Services, Windows 2.0.6 Excel 2002 Edition). Statgraphics^®^ Plus 4.1. Professional Version (Statistical Graphics Corp) was used for the validation of bioanalytical methods and linearity calculations. 

##  Results

###  Development and validation of bioanalytical methods

The methods for identification and quantification of stavudine, lamivudine and zidovudine were developed in plasma for the pharmacokinetic studies and in HBSS for permeability tests. To validate the methods, calibration curves were made by preparing ten different dilutions of the standards (five replicates per concentration) in HBSS or drug free plasma. The linearity was evaluated in the range of 0.25 to 6.0 g/mL for d4T and 3TC, and 0.25 to 8.0 g / mL for AZT. The standards prepared in HBSS and plasma were stable for more than 90 days at -75° +/- 5° C. All methods met the criteria: linearity (R² ≥0.8), precision (CV <15%), accuracy (CV <15%) recovery (> 80%), lower limit of detection and quantification (Table 1).

**Table 1.** Bioanalytical methods validation.

Parameter	Hanks Balanced Salt Solution			Human plasma		
	Stavudine	Lamivudine	Zidovudine	Stavudine	Lamivudine	Zidovudine
Linearity	y= 1.061x+0.019	y= 0.971x-0.025	y= 0.814x+0.085	y= 1.055x-0.022	y= 0.942x-0.055	y= 0.567x-0.016
	R²= 0.9990	R²= 0.999	R²= 0.9901	R²= 0.9958	R²= 0.995	R²= 0.9993
Precision (Coefficient of variation %)	2.07	1.92	2.36	2.76	3.48	1.45
Accuracy (Relative error %)	0.32	0.14	1.25	4.48	0.23	0.15
Low limit of detection (ug/mL)	0.010	0.005	0.023	0.048	0.025	0.003
Low limit of quantitation (ug/mL)	0.026	0.014	0.075	0.111	0.078	0.005
Recovery (%)	99.05	99.87	97.12	95.61	99.21	99.65

### Dissolution profile

The kinetics of dissolution were determined following pharmacopeia parameters at 10, 15, 20, 30, 45, 60 min in 12 independent tests. The mean value at every time point was used to calculate the logarithmic linear regression and the mathematical model of the dissolution profiles, where *y* is the percentage of dissolved drug and *x* is time:

Stavudine: y = 0.0097ln(x) + 3.6246 

Lamivudine: y = 0.0345ln(x) + 5.0272 

Zidovudine: y = 0.0975ln(x) + 5.6808 

Similar interpolation accumulation periods in Caco-2 monolayers and cells accumulated area under the curve (AUC) pharmacokinetics in humans and rabbits was performed (Table 2).

**Table 2.** Dissolutions profiles and accumulation percent in Caco-2, rabbits and humans.

Drugs		Time (h)					
		0.17	0.25	0.33	0.50	0.75	1.00
Zidovudine	Dissolution	83.6	86.9	89.4	93.0	96.8	99.5
	Accumulate Caco-2	0.95	2.10	3.63	7.73	16.39	27.89
	Accumulate AUC Rabbit	2.37	5.04	8.22	15.24	26.07	36.19
	Accumulate AUC Human	2.7	5.8	9.55	18.07	31.48	44.02
Lamivudine	Dissolution	94.8	96.2	97.1	98.5	99.9	100.9
	Accumulate Caco-2	0.74	1.67	2.94	6.54	14.53	25.60
	Accumulate AUC Rabbit	0.75	1.64	2.82	5.86	11.64	18.33
	Accumulate AUC Human	0.17	0.38	0.67	1.46	3.12	5.27
Stavudine	Dissolution	92.4	92.7	93.0	93.4	93.7	94.0
	Accumulate Caco-2	0.76	1.70	3.00	6.64	14.68	25.78
	Accumulate AUC Rabbit	3.64	7.57	11.89	20.66	32.87	43.4
	Accumulate AUC Human	2.23	4.72	7.63	13.94	23.53	32.48

###  Permeability assays in Caco-2 cells

The parameters for the permeability assays in cell monolayers were standardized. In addition to test the integrity of the monolayers by TEER, markers for transcellular and paracelullar transport were used, Metoprolol (high permeability) and Mannitol (low permeability), respectively. The Papp of metoprolol was 47.2x10^-6 ^cm/seg (SD ± 5.5x10^-6^, n= 5). On the other hand, it was not possible to determine the Papp for mannitol since the concentrations in the recipient compartment were below the limit of detection. The determination of the changes in concentration in the receiver compartment at 15, 30, 45, 60, 90 and 120 min in ten independent tests allowed us to evaluate the indicators of diffusion (Table 3). The data obtained from first order kinetics were adjusted by least squares regression, with a correlation coefficient >0.99, where *y *is the concentration (µg/mL) and *x *is the time (h): 

Stavudine: y = 0.9548Ln(x) + 2.1457 

Lamivudine: y = 0.9655Ln(x) - 0.0574 

Zidovudine: y = 0.8391Ln(x) + 1.6946

These equations were applied to the correlation with dissolution at one hour and the pharmacokinetics in humans and rabbits.

**Table 3.** Diffusion parameters in Caco-2 cells monolayers.

Parameter*	Units	Stavudine	Lamivudine	Zidovudine
*P*app x10^-6^	cm/s	6.4 ± 1.8	7.0 ± 2.0	20.3 ± 4.7
Flujo (*J*)	µg/cm^2^/min	0.081 ± 0.003	0.041 ± 0.005	0.063 ± 0.004

*Mean ± SD (n= 10)

Papp= Apparent permeability coefficient

###  Pharmacokinetics in rabbits

The doses administered to the animals (d4T 0.60 mg/Kg, 3TC 2.13 mg/Kg y AZT 4.14 mg/Kg) were normalized with respect to the doses for human volunteers, to calculate the proportionality between them (rabbit/human). These factors were: d4T 14, 3TC 9.78 y AZT 10.06. The plasma concentration results were used to evaluate the absorption and elimination phases in the logarithm of the concentration versus time curve, where *y *is the concentration of the drug in plasma and *x* is the sampling time, in the elimination phase (R² >0.97), then:

Stavudine: y = -0.6906x + 1.3588 

Lamivudine: y = -0.7271x + 4.2558

Zidovudine: y = -0.6206x + 2.9383

**Figure 1.**
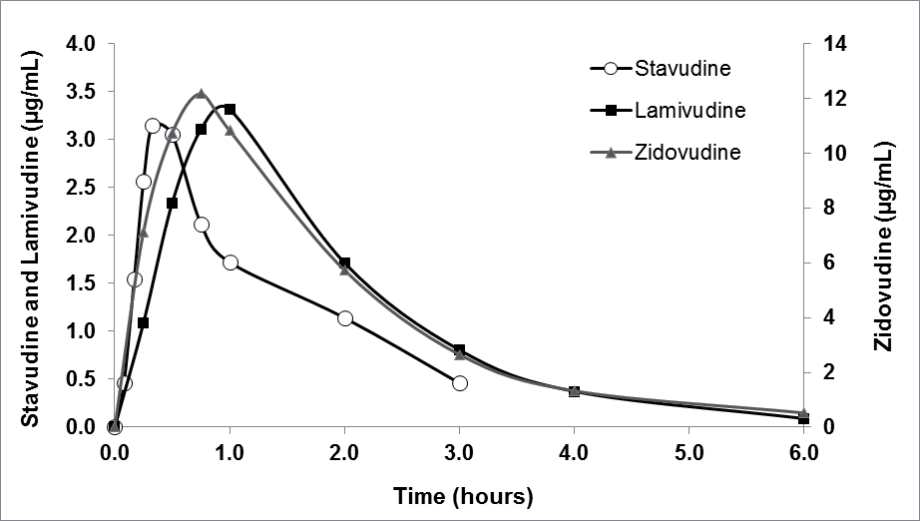
Pharmacokinetics in animal models.

The simulated data obtained using these equations, adjusted by the factor corresponding to human doses, were used in the non-compartmental pharmacokinetic analysis to test the partial and accumulative areas under the curve (AUC_0-t_, AUC_Acum_) according to the Wagner-Nelson (Fig. 1), and calculated by:

Cp= (F * Dosis * Ka / Vd * Ka - Ke )(e-^ke*t ^- e -^Ka ke*t)^

Expressed as: 

Cp = I (e-^ke*t ^- e -^Ka*t)^

The main pharmacokinetic parameters are described in Table 4. The plasma concentration of the three antiretrovirals reached a maximum value rapidly and later decreased to reach baseline values 6 h after treatment. These results showed the rapid absorption and high permeability of the three APIs. AZT reached the highest bioavailability of the three antiretrovirals. 

**Table 4.** Pharmacokinetics parameters in the animal model and humans.

Product		Rabbit Pharmacokinetic			Human Pharmacokinetic		
		Zidovudine	Lamivudine	Stavudine	Zidovudine	Lamivudine	Stavudine
C_max_	µg/mL	12.17	3.31	3.15	2.12	0.94	0.73
T_max_	h	0.75	1.00	0.33	0.33	1.00	0.50
T_1/2_	h	1.12	0.95	1.00	0.72	2.68	1.37
AUC_ (0-t)_	h*µg/mL	25.17	6.87	4.38	2.16	3.51	1.67
AUC_(0-∞)_	h*µg/mL	26.00	6.99	4.90	2.16	3.51	1.67
AUMC_ (0-t)_	µg-h*h/mL	41.34	11.62	4.97	3.02	10.41	3.02
AUMC_(0-∞)_	µg-h*h/mL	47.71	12.53	7.10	3.02	10.41	3.02

C_max_ = maximum concentration

T_max_ = time required to reach the maximal concentration

AUC= area under the curve in time

AUMC= Area under the first moment curve

### Pharmacokinetics in humans 

**Figure 2.**
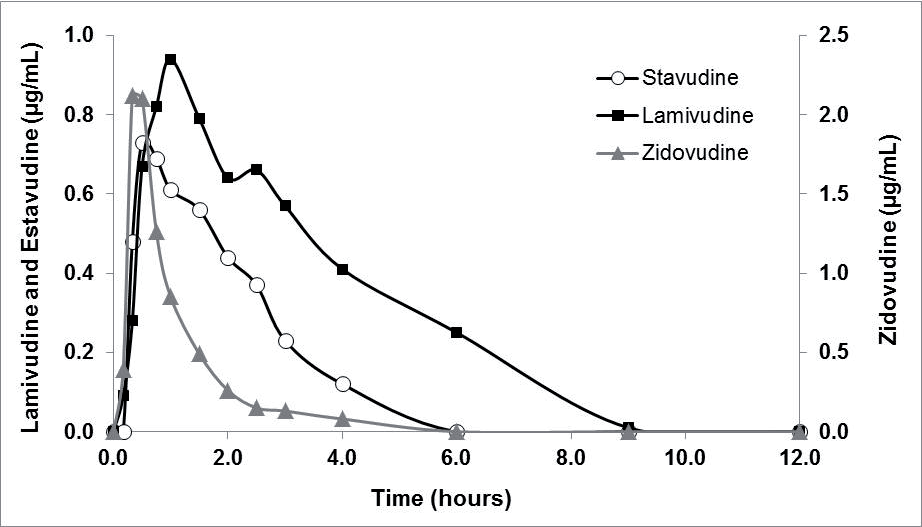
Pharmacokinetics in humans.

The pharmacokinetic profiles were the main reference parameter for the *in vitro-in vivo* correlation (Table 4). The plasma profile of concentration versus time after the administration of a single oral dose of stavudine (40 mg) or lamivudina (150 mg)/zidovudine (300 mg) is shown in Figure 2. 

The linear regression of the logarithm of the plasma concentration versus time, provided the mathematical models for the elimination phase (R² >0.97). The three antiretroviral tested were rapidly absorbed after oral administration (Tabla 4). 

Stavudine: y = -0.5046x + 0.0649

Lamivudine: y = -0.2595x + 2.4725

Zidovudine: y = -0.9678x + 0.9018

### *In vitro-in vitro* correlations

**Dissolution versus permeability. **The percentages of concentration changes between zero and one hour (maximum time of dissolution) and the changes in concentration in the recipient compartment in the permeability test were compared (Table 2). The linear regression mathematical models showed that the dissolution data are optimal to predict permeability by diffusion (R² >0.80). 

Stavudine: y = 14.581x - 1,350, R²= 0.8368

Lamivudine: y = 3.8844x - 371.6, R²= 0.8401

Zidovudine: y = 1.6163x - 138.14, R²= 0.8655

Where *y *is the AUC accumulative percentages in Caco-2 cells and *x *is the percentage of dissolution. These functions can be implemented to predict the quality of drugs in terms of permeability and the quality of excipients from different sources during the manufacturing process.

### *In vivo-in vivo* correlations

**Figure 3.**
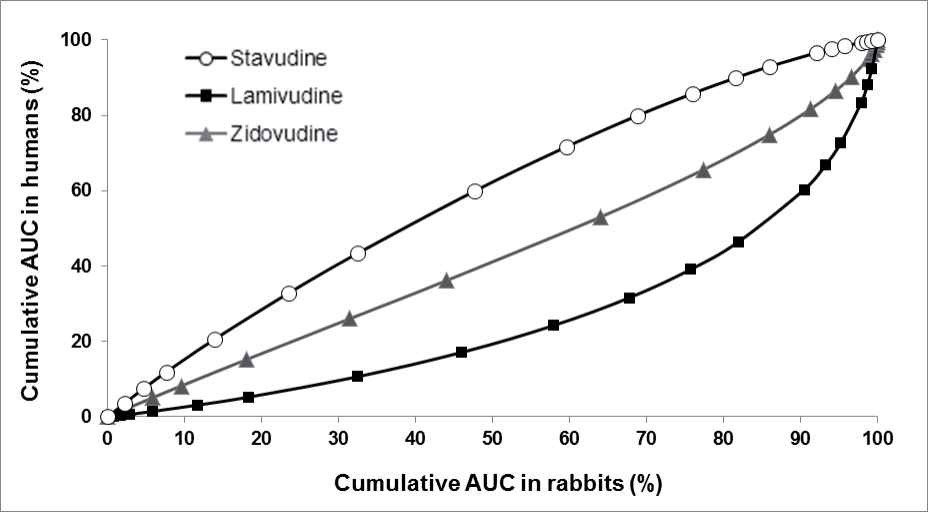
Correlation cumulative AUC in humans versus animal model.

**Rabbit vs. humans. **The cumulative areas under the curve were correlated and a statistical analysis by least squares was applied (Fig. 3). A high correlation between the pharmacokinetic profiles in humans and in the animal model was observed (stavudine R^2^= 0.987, lamivudine R^2^= 0.885 and zidovudine R^2^= 0.989). The AUC_0-t _for zidovudine in both species was proportional to the dose. However, for lamivudine even though the ratio rabbit/human was ten, the AUC_0-t _only exceeded the value in humans by a factor of two. For stavudine, the dose ratio was 15 times higher for rabbits than humans, but the AUC_0-t _in rabbits was only two and a half times the AUC_0-t _in humans. 

The C_max_ values presented the largest interspecies differences regarding the fraction of drug absorbed, therefore the use of this parameter to predict the behavior of these drugs is not recommended. The time to reach the maximum concentration (T_max_) of zidovudine was two times higher in humans than in rabbits (0.75 h and 0.33 h, respectively), while for lamivudine and stavudine the values were similar (Table 4). Consequently, for these drugs T_max _in rabbits was directly related to the expected results in humans. The comparison of the *in vivo* results, in rabbits and humans for AUC_0-t_, was based on the plasma profiles and patterns of elimination in the curve of the logarithm of concentration versus time for simulated changes in the same periods (Wagner Nelson Method). 

### Correlation of the accumulative percentages of AUC in humans versus rabbits

The mathematical models of the linear correlation and the corresponding factors (R^2)^ to the least squares regression were:

Stavudine: y = 0.9709x + 7.6027, R² = 0.987

Lamivudine: y = 0.8562x - 9.3888, R² = 0.885

Zidovudine: y = 0.9838x - 3.4354, R² = 0.988

*X* values represent cumulative AUC in rabbits interpolated for cumulative AUC in humans at the same period for equivalent doses. The correlation factors demonstrated the ability of the rabbit species to predict bioavailability in humans due to the amount of absorbed and accumulated drug over time, with a probability close to or greater than 90%. These mathematical models exceed the biological variability analysis and inter-species differences because each subject responded comprehensively combining the physiological properties of the animal and the human subject under test conditions.

### *In vitro-in vivo* correlations

**Dissolution versus AUC_0-t_ in Humans**. The dissolution profiles and pharmacokinetics in animals and humans were compared (Table 2). Direct, exponential correlation between the percentage of the drug dissolved *in vitro* and cumulative AUC in humans was found (R^2^> 0.99).

Stavudine: y = 1.4842^e0.5376x^

Lamivudine: y = 0.0902^e0.6906x^

Zidovudine: y = 1.7444^e0.5619x^

In these equations *x* represents dissolution percentage values and *y *represents* in vivo* projected AUC for the same period. The Pharmacokinetic data in the animal model also showed satisfactory results with exponential models and correlation factors greater than 0.98. A direct exponential relationship between the percent of dissolution and the amount of drug available in the blood plasma was used to predict the effectiveness of the absorption processes based on the data of *in vitro* dissolution percentages.

**Figura 4.**
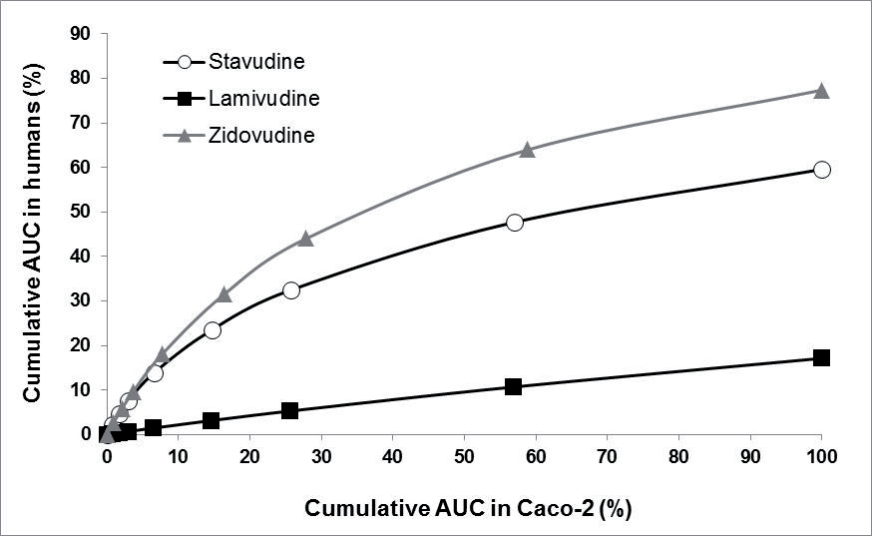
Correlation *in vitro* permeability versus *in vivo* absorption.

**Permeability versus AUC in humans. **Cumulative AUC values in the Caco-2 system were correlated with AUC values *in vivo* in the first two hours since the permeability test was performed for two hours. A directly proportional relation for lamivudine was found, while for stavudine and zidovudine the relation was logarithmic (Fig. 4). The following functions expressed the direct relationship between the percentage of transferred drug in Caco-2 cells and the post-administration cumulative AUC percentage.

Stavudine: y = 11.820ln(x) - 2.3697 R²= 0.9305 

Zidovudine: y = 16.488ln(x) - 7.2008 R²= 0.9332 

Lamivudine: y = 0.172(x) + 0.3678 R²= 0.9961 

The results of the comparisons in the animal model were equally satisfactory with exponential functions and correlation factors greater than 0.90. 

### Multiple Linear Regressions

**Pharmacokinetics in humans vs dissolution percent and permeability in Caco-2 cells**. The final interpretation of the relationship between *in vitro* results (dissolution percent and the amount of drug diffused through Caco-2 monolayers), and the amount of drug accumulated over time in humans was performed by multiple linear regressions with a confidence interval of 95% (*p *<0.05) for the following functions:

Independent variables: Dissolution percent and cumulative AUC in Caco-2.

Dependent Variable: cumulative AUC in humans at the first hour post-absorption.

Stavudine: R^2^ 0.986

AUC_AcuHum _= 2.681 + (0.0149 ∗ Dissolution percent) + (1.170 ∗ AUC_AcuCaco-2_) 

Lamivudine: R^2^ 0.999

AUC_AcuHum _= -1.713 + (0.0185 ∗ Dissolution percent) + (0.202 ∗ AUC_AcuCaco-2_) 

Zidovudine: R^2^ 0.991

AUC_AcuHum _= 2.255 + (0.0232 ∗ Dissolution percent) + (1.488 ∗ AUC_AcuCaco-2_) 

Similar results with correlations greater than 0.97 were obtained from the multiple regressions, considering the same variables in the animal model.

## Discussion

Regulations require assessing the quality of drugs through *in vivo* bioavailability and bioequivalence tests in healthy adult humans. This study proved the validity of applying surrogate *in vitro* tests to reduce the costs and ethical risks associated to the inclusion of humans in research, as long as solid scientific evidence support the substitution. These tests are based on the solubility and permeability properties of the active pharmaceutical ingredients, according to the biopharmaceutics classification system (BCS) 3. Stavudine, lamivudine and zidovudine are Type I compounds, highly soluble and highly permeable 8. Type I drugs in immediate release oral products, can be waived from human studies, however, currently their quality is demonstrated by *in vivo* bioavailability and bioequivalence tests.

### Physicochemical assessment and dissolution test

The analyzed products meet the quality specifications for drug content, dose uniformity and dissolution profile according to the United States pharmacopeia (USP34, NF29, 2011) and related documents 9-13. In less than 20 min 99.0% of lamivudine, 91.2% of zidovudine and 93.5% of stavudine were dissolved. The solubility test supported the recommendation of exemption from *in vivo* bioequivalence studies for dosage forms of immediate release like stavudine, lamivudine and zidovudine 14,15. The dissolution profile confirmed that 81.8% of zidovudine was released from the tablets in the first 10 minutes and 93.4% of lamivudine. The capsules of stavudine released more than 90.0% of the API in the first 15 min.

### Permeability tests in Caco-2 cells

Metoprolol and mannitol were used as high and low permeability standards respectively and the results were concordant with other studies and FDA guidelines 16,17. This support the suitability of our *in vitro* system to discriminate between high and low permeability compounds. The results obtained when testing stavudine and lamivudine were very similar and fit the definition of highly permeable drugs. The permeability of zidovudine was three times higher than the permeability of stavudine and lamivudine which is related to its physicochemical properties of lipophilicity. This methodology is in compliance with the requirements of the guidelines for *in vitro* test for the evaluation of solubility and permeability properties established in the BCS and other publication in the field 18-21. The functionality of the Caco-2 system was confirmed by calculating the apparent permeability coefficient (Papp), permeability coefficient (P) and flux coefficient (J).

### Pharmacokinetic studies

The rabbit animal model proved to be an efficient way to evaluate absorption process according to biological and biochemical properties that are similar to humans, therefore supporting its use in pre-clinical research 22,23. The guidelines for good laboratory practices and use of animals in bioavailability and bioequivalence studies were followed 24-29. The three drugs reached the maximum plasma concentration in an hour or less, being around 20 min for stavudine. The half-life time in rabbits for lamivudine was 0.95 h, shorter than the experimental value in humans (2.68 h), it was slightly longer for zidovudine 1.12 h (0.72 h) and shorter for stavudine 1.0 h (1.37 h).

The human studies were taken as references for quality in terms of plasma concentration and AUC for a period of 12 h 30-33. The evaluated parameters were: maximum concentration (C_max_), area under the curve in time (AUC_0-t_) and time required to reach the maximal concentration (T_max_).

This study demonstrated the bioavailability of the dosage forms zidovudine 300 mg/lamivudine 150 mg tablets and stavudine 40 mg capsules manufactured by Humax Pharmaceutical S.A, and the compliance with quality standards according to the dissolution properties, drug content and other physicochemical properties described for these dosage forms. The diffusion and permeability capacity of the compounds was confirm using the cell monolayer model and a direct correlation with the pharmacokinetic of absorption in the animal model and humans was found. The analytical methods and validated procedures applied to *in vitro* tests demonstrated the direct correlation with the results obtained in the pharmacokinetic studies, with correlation coefficients greater than 0.85. 

## Conclusions 

This study achieve the goal to implement analytical methodologies, that are commonly applied in the discovery and development of new drugs, to measure the physical and biological properties of the compounds that correlate with the bioavailability studies in humans, according to the principles of IVIVC. In all the comparisons the linear regression by the least square method provided correlation factors close or greater than 0.9, the tested comparisons were:

*in vitro-in vitro*: Permeability-Dissolution

*in vitro-in vivo*: Dissolution-Pharmacokinetics

*in vitro-in vivo*: Permeability-Pharmacokinetics

*in vivo-in vivo*: Pharmacokinetics Animal Model-Human

The results of this study support the proposal to revise the regulatory requirements in force in Colombia, related to *in vivo* tests like bioequivalence studies for immediate release products with APIs classified as BCS-I, specifically antiretroviral nucleoside reverse transcriptase inhibitors.

It is proposed to improve the capacity of the pharmaceutical industry to standardize *in vitro* and *in vivo* (rabbits) methods, complementary to the dissolution tests, in order to validate the results between them and to give strength and power, which is essential for quality assurance for *in vivo *behavior.
